# Prediction of the Long-Term Performance of an Existing Warm Recycled Motorway Pavement

**DOI:** 10.3390/ma16031005

**Published:** 2023-01-21

**Authors:** Lorenzo Paolo Ingrassia, Sara Spadoni, Gilda Ferrotti, Amedeo Virgili, Francesco Canestrari

**Affiliations:** Department of Civil and Building Engineering and Architecture (DICEA), Università Politecnica delle Marche, Via Brecce Bianche, 60131 Ancona, Italy

**Keywords:** warm mix asphalt (WMA), reclaimed asphalt pavement (RAP), full-scale field trial, KENPAVE, FlexPAVE, viscoelastic continuum damage (VECD), pavement design

## Abstract

Warm mix asphalt (WMA) technologies allow the production, lay-down and compaction of asphalt mixtures at reduced temperatures and the use of higher amounts of reclaimed asphalt pavement (RAP) with respect to conventional hot mix asphalt (HMA), leading to significant environmental benefits and energy savings. However, limited data is available on the long-term performance of such pavements. The objective of this study was to predict the long-term performance of an existing warm recycled motorway pavement (made with WMA mixtures containing RAP) constructed in 2016 in central Italy, along with the corresponding hot recycled pavement (made with HMA mixtures containing RAP). For this purpose, cores were taken from the pavements in 2022 to investigate the binder and base courses through dynamic modulus and cyclic fatigue tests, according to the simplified viscoelastic continuum damage (S-VECD) testing approach. The results of the tests were used to predict the service life of the pavements using two pieces of software, KENPAVE and FlexPAVE, based respectively on the elastic design method and the viscoelastic design method in the presence of damage. The FlexPAVE results indicated that the expected service life of the WMA pavement is much longer than that of the HMA pavement, mainly because the WMA mixtures have better damage properties than the HMA mixtures. Conversely, the KENPAVE simulations predicted a similar service life for the two pavements, highlighting the impossibility of the elastic method to catch the actual contribution of high-performance non-standard materials. The promising outcomes of the FlexPAVE simulations further encourage the application of warm recycled pavements.

## 1. Introduction

In the last decade, warm mix asphalt (WMA) technologies have gained growing attention in pavement engineering, because they allow the production, lay-down and compaction of asphalt mixtures at reduced temperatures, about 40 °C lower than conventional hot mix asphalt (HMA). Such significant temperature reduction leads to a series of benefits for the environment and for human health, thanks to the lower toxic emissions, odors and fumes generated. Energy savings can also be achieved compared to HMA, which makes such technologies even more attractive nowadays given current energy costs [1,2,3,4,5,6,7,8]. Moreover, the typical working temperatures adopted for WMA imply lower binder aging, thus allowing the use of higher amounts of reclaimed asphalt pavement (RAP) in recycled asphalt mixtures with respect to HMA, in full agreement with the principles of the circular economy [9,10].

From a mechanical point of view, preliminary laboratory [11,12,13,14] and field [15,16,17,18,19] results are promising and suggest that a performance improvement can be obtained with respect to conventional HMA. However, also due to the relatively recent development of WMA technologies, limited data is currently available in the literature on the long-term performance of warm recycled asphalt pavements, especially under the effect of motorway heavy traffic.

In the absence of comprehensive historical data, one way to predict the service life of a pavement is to use ad hoc design tools [20]. The most used tools model the pavement as a linear elastic multi-layer subjected to a static load, thus allowing the calculation of stresses and strains in critical points within the pavement. The main necessary inputs are the characteristics of the applied static load, the pavement structure and the representative stiffness moduli of the pavement courses in the seasons of the year (e.g., winter, spring, summer, autumn). One of the most common elastic-based design tools is KENPAVE software [21].

FlexPAVE software is a more advanced design tool, which allows the modeling of the pavement as a multi-layered viscoelastic structure subjected to moving loads and thermal effects. The long-term performance is then predicted in terms of damage evolution within the pavement over time with the finite element method [22,23]. To model the asphalt mixtures as viscoelastic materials subjected to damage, their properties should be determined through dynamic modulus and cyclic fatigue tests, based on the simplified viscoelastic continuum damage (S-VECD) testing approach that has its theoretical foundation in the viscoelastic continuum damage (VECD) theory [24]. To assess the damage induced by thermal effects, FlexPAVE is integrated with the enhanced integrated climatic model (EICM) database, which includes one-year daily temperature variations for about 600 locations in the United States. Moreover, the moving nature of the traffic loads is taken into account through the representative speed of the vehicles.

**Figure 1:**
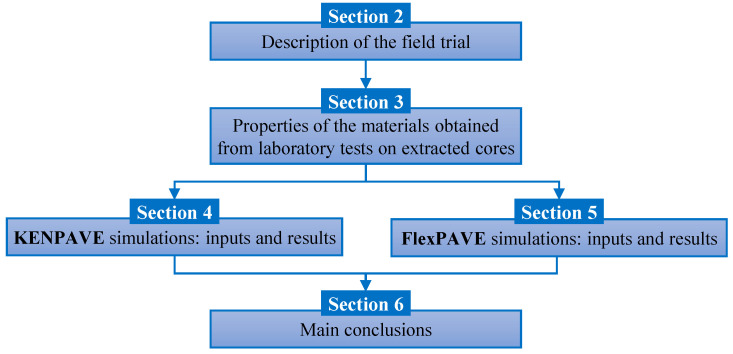
Structure and main contents of the paper.

Based on this background, the objective of this study was to predict the long-term performance of an existing motorway field trial constructed in 2016 in central Italy, which included a warm recycled pavement (i.e., made with WMA mixtures containing RAP) and the corresponding hot recycled pavement (i.e., made with conventional HMA mixtures containing the same percentages of RAP). For this purpose, cores were taken from the two pavements after about six years of in-service life. The binder and base courses were then investigated through dynamic modulus and cyclic fatigue tests, based on the S-VECD testing approach. The results of the tests were used to predict the service life of the two pavements using KENPAVE and FlexPAVE (v1.1). The structure and the main contents of the paper are summarized in the flow chart in Figure 1.

## 2. Description of the Field Trial

**Figure 2:**
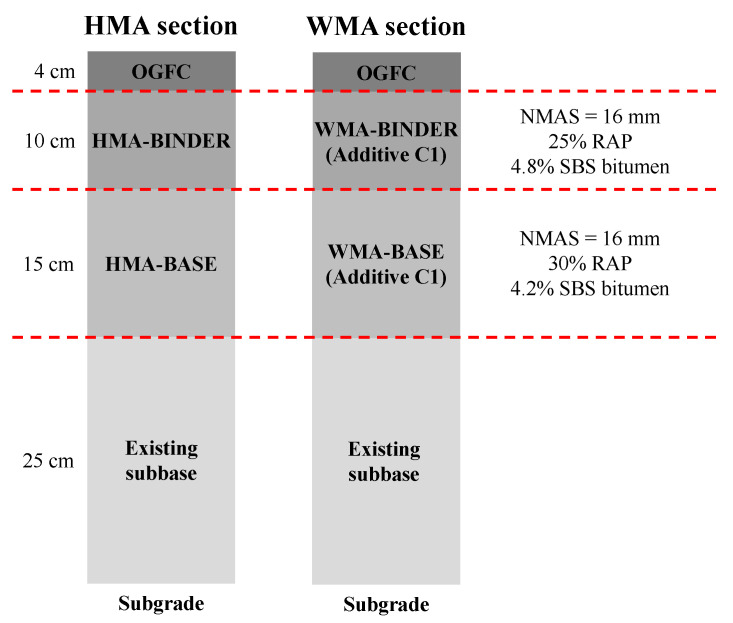
Cross-section of the HMA and WMA pavements.

The studied field trial was constructed in 2016 along the A1 motorway in central Italy. The field trial included two consecutive 200 m straight sections, one constructed with HMA mixtures and one with WMA mixtures. The construction activities consisted of the full-depth milling and reconstruction of the asphalt courses in the slow lane. Both trial sections presented the same pavement structure, i.e., a 4 cm open-graded friction course (OGFC), a 10 cm binder course and a 15 cm base course over the existing cold recycled subbase course with a nominal thickness of 25 cm (Figure 2). Specifically, the subjects of this study are mainly the binder and base courses.

The binder course mixtures (both HMA and WMA) were characterized by a nominal maximum aggregate size (NMAS) of 16 mm and contained 25% by aggregate weight of unfractioned 0/14 mm RAP and 4.8% by aggregate weight of total bitumen content (virgin bitumen plus bitumen from RAP). The base course mixtures were characterized by a NMAS of 16 mm and contained 30% unfractioned 0/14 mm RAP and 4.2% total bitumen content. In all cases, the virgin bitumen and RAP bitumen were modified with styrene–butadiene–styrene (SBS) polymers (3.8% polymers by bitumen weight).

The WMA mixtures were produced with a chemical additive (C1) available on the market, mainly composed of ammine substances acting as adhesion enhancers and surfactants. C1 is a viscous liquid at 25 °C with a density of 1.0 g/cm^3^ and is characterized by a pour point of −8 °C and a flash point higher than 140 °C. Its dosage was chosen based on the producer's recommendations. Thanks to the use of the additive C1, the production and compaction temperatures of the WMA mixtures were respectively equal to 130 °C and 120 °C, i.e., 40 °C lower than the HMA mixtures (produced at 170 °C and compacted at 160 °C).

Additional information on the field trial and the materials used, as well as historical performance data, can be found in previous publications [25,26].

## 3. Properties of the Materials

**Figure 3:**
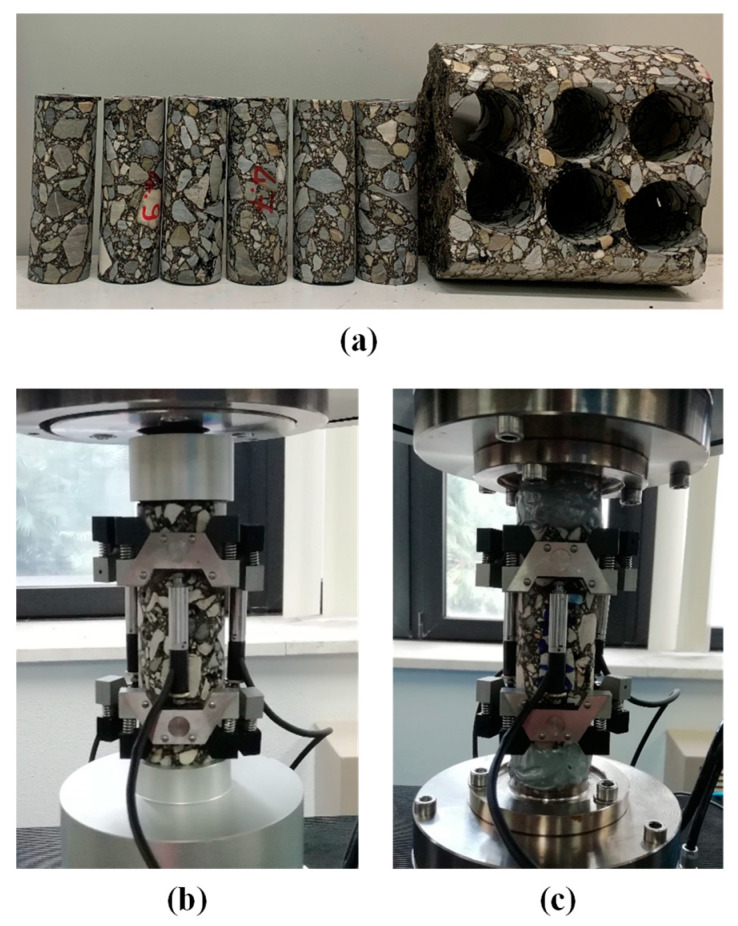
Experimental investigation: (**a**) testing specimens horizontally cored from the base course, (**b**) setup of the dynamic modulus tests, (**c**) setup of the cyclic fatigue tests.

To study the properties of the materials, 150 mm diameter cores were taken from the trial sections in the middle of the lane. Afterwards, testing specimens with 38 mm diameter and 110 mm height were horizontally cored from the binder and base courses [27], as shown in Figure 3a. The viscoelastic properties of the materials (norm of dynamic modulus $\left|E^{*}\right|$ and phase angle *δ*) were investigated through dynamic modulus tests (Figure 3b), performed at different temperatures (4, 20, 40 °C) and frequencies (0.1, 0.5, 1, 5, 10, 20 Hz) based on the standard AASHTO TP 132 [28]. The tests were carried out in compression configuration, keeping the axial strain between 50 and 75 με. Three specimens were tested for each mixture. The experimental data (specifically, the storage modulus data, $E1=\left|E^{*}\right|\cdot \cos\delta$) were then shifted at the reference temperature of 21.1 °C (i.e., 70 °F) and fitted with the 2S2P1D model [29]. The fatigue behavior of the materials from the trial sections was investigated through cyclic fatigue tests (Figure 3c) in direct tension configuration at a frequency of 10 Hz and a temperature of 21 °C, according to the standard AASHTO TP 133 [30]. At least three specimens were tested for each mixture, considering different strain levels.

The analysis of the results, based on the S-VECD approach, allowed the obtention, for each mixture, of the so-called damage characteristic curve, i.e., a relationship between the damage level (*S*) and the material integrity, expressed in terms of pseudo-stiffness (*C*). Such a relationship describes the intrinsic damage properties of the material (i.e., it is independent of the boundary and testing conditions) and can be expressed through a power function law, as in Equation (1) [31]. The *D^R^* failure criterion was considered to define the material failure, where *D^R^* is another material-specific property that represents the average reduction in pseudo-stiffness up to failure (Equation (2)) and is a measure of the mixture toughness [32].

(1) $$C=1-C_{11}\cdot S^{C_{12}}$$

(2) $$D^{R}=\frac{\int_{0}^{N_{f}}\left(1-C\right)dN}{N_{f}}=\frac{Cum\left(1-C\right)}{N_{f}}$$

where *C*_11_ and *C*_12_ are fitting coefficients; *N_f_* is the number of cycles to failure, defined as the cycle in which the product of peak-to-peak stress and cycle number reaches a maximum value during the test [30].

**Figure 4:**
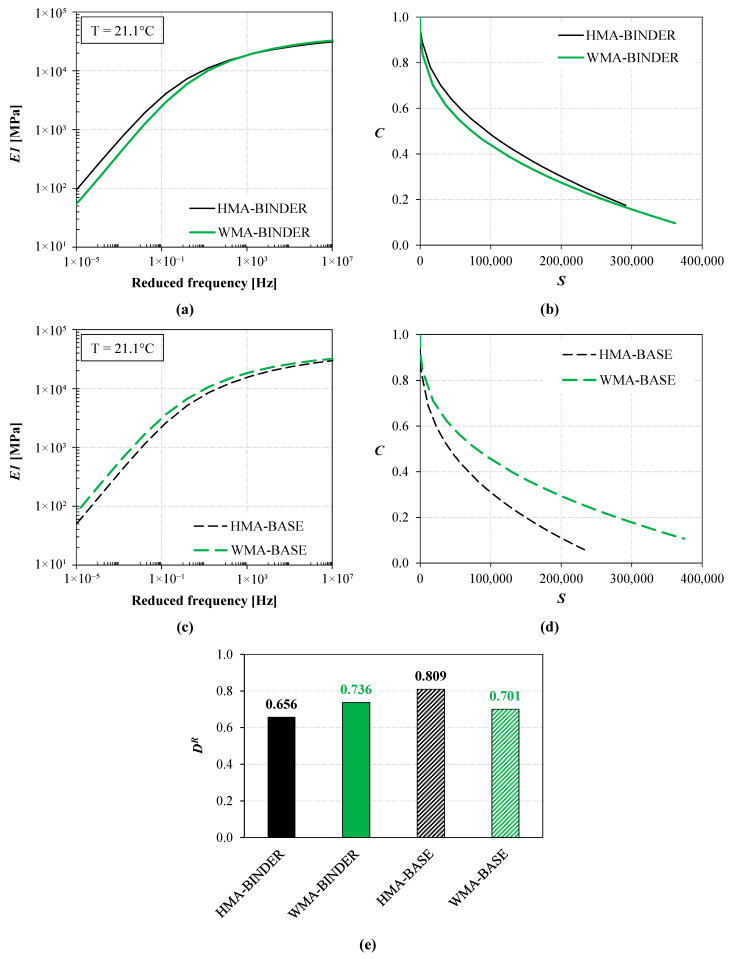
Properties of the materials from the field trial: (**a**) storage modulus master curve (2S2P1D model) and (**b**) damage characteristic curve of the binder course mixture, (**c**) storage modulus master curve (2S2P1D model) and (**d**) damage characteristic curve of the base course mixture, (**e**) *D^R^* values.

The results of the laboratory tests are shown in Figure 4, whereas the average air void content obtained for each mixture (saturated surface dry method [33]) is reported in Table 1. As for the binder course, the HMA mixture is characterized by a higher stiffness with respect to the WMA mixture in a wide range of reduced frequencies (Figure 4a), despite their comparable air voids (Table 1). These results can be ascribed to a higher RAP oxidation during production, laying and compaction and/or the faster in-service aging experienced by the HMA mixture. In addition, Figure 4b shows that, for the binder course, the damage characteristic curve of WMA presents a lower pseudo-stiffness value and a higher amount of damage at failure compared to HMA, which suggests a better damage tolerance for the WMA mixture (i.e., postponed failure). Finally, Figure 4e indicates that the WMA mixture (*D^R^* = 0.736) is tougher than the HMA mixture (*D^R^* = 0.656), i.e., it can absorb more energy before failure. In the case of the base course, the WMA mixture is stiffer than the HMA mixture (Figure 4c) due to its lower air void content (Table 1). As a consequence of the stiffness properties, the damage characteristic curve of the HMA mixture is much lower than that of the WMA mixture (Figure 4d). At the same time, the toughness of the WMA mixture (*D^R^* = 0.701) is somewhat penalized by its lower air voids compared to the HMA mixture (*D^R^* = 0.809). The difference in the air voids observed in the base course for the two mixtures could be a result of the worse compactability of the HMA mixture. More details on the characterization of the materials from the field trial and a deeper discussion about their properties can be found in a previous publication [34].

**Figure 5:**
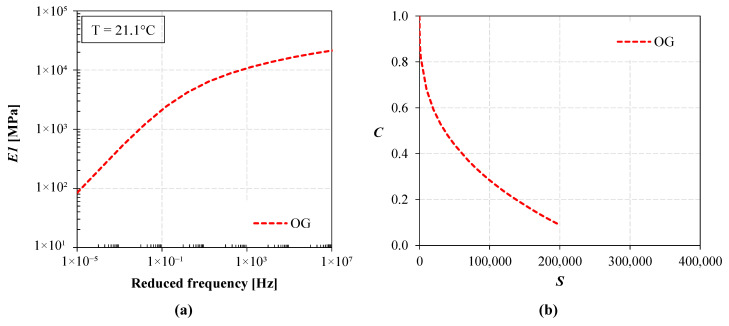
Properties of the OG mixture: (**a**) storage modulus master curve (2S2P1D model), (**b**) damage characteristic curve.

As mentioned in Section 2, the existing field trial includes a 4 cm OGFC, which could not be studied with the S-VECD approach due to its limited thickness. To also include this course in the pavement simulations, the results obtained in a previous investigation [35] for an open-graded mixture without RAP (coded as OG) were considered. The OG mixture was characterized by an NMAS of 14 mm and contained 5.1% by aggregate weight of SBS polymer-modified bitumen (3.8% polymers by bitumen weight) and 0.3% by aggregate weight of cellulose and glass fibers (to avoid drain-down issues). The mixture was produced in an asphalt plant at 170 °C and then compacted in the laboratory at 160 °C with the gyratory compactor to produce samples with 150 mm diameter and 180 mm height. One testing specimen with 100 mm diameter was cored from the inner portion of the gyratory-compacted sample [36]. For the OG mixture, dynamic modulus tests were performed at various temperatures (in the range 4 ÷ 40 °C) and frequencies (in the range 0.1 ÷ 10 Hz) keeping the axial strain between 75 and 125 με, according to AASHTO T 378 [37]. The cyclic fatigue tests were carried out at 10 Hz and 21 °C considering different strain levels, according to AASHTO T 107 [38]. The results of these tests are reported in Figure 5. The average air void content of the test specimens of the OG mixture was 19.3% (geometric method [33]).

## 4. KENPAVE Simulations

### 4.1. Input Data

Sixteen different KENPAVE simulations were performed overall. For each trial section (HMA and WMA), the conditions of intact subbase and cracked subbase were considered. The intact condition simulates the initial phase of the in-service life of the cold recycled subbase, i.e., when it behaves as a bound material. The cracked condition simulates the second phase of the in-service life of the layer, during which the subbase behaves similarly to a granular unbound material [39]. Moreover, to indirectly account for the temperature dependence of the asphalt courses, four simulations were performed for each section and subbase condition by splitting the calculation into four seasons (winter, spring, summer and autumn) and considering the average temperature of the pavement (T_AC_) in each season. The values of T_AC_ (reported in the heading of Table 2) were calculated at a depth equal to one-third of the overall thickness of the asphalt courses (i.e., about 10 cm) based on the average seasonal air temperatures at the field trial location, according to [40]. Then, the stiffness moduli of the asphalt courses (summarized in Table 2) were obtained from the materials’ master curves (Figure 4a,c and Figure 5a) considering a frequency of 15 Hz, which corresponds to the typical speed of motorway heavy traffic of 90 km/h [41,42]. Specifically, the time–temperature superposition concept was applied to convert the frequency of 15 Hz at the pavement temperature T_AC_ into the equivalent reduced frequency at the reference temperature of the master curves (21.1 °C).

The pavement structure studied in the simulations was the same as the existing field trial, i.e., a 4 cm OGFC, a 10 cm binder course, a 15 cm base course and a 25 cm subbase over the subgrade (see Figure 2). In the absence of more specific data, the same stiffness was assumed for the subbase in the four seasons (Table 2). In the simulations with intact subbase, a stiffness modulus of 1200 MPa was considered, based on the outcomes of previous falling weight deflectometer (FWD) tests on the pavement [25]. In the simulations with cracked subbase, the stiffness modulus was assumed to be equal to 400 MPa, which is a typical value for granular materials. For the subgrade, a stiffness modulus of 100 MPa was considered (Table 2), based on the outcomes of FWD tests [25].

The traffic loads were defined considering a reference 120 kN single axle with twin wheels (spaced by 30 cm). The tire inflation pressure was set as equal to 800 kPa (typical value for heavy vehicles), which resulted in a circular tire–pavement contact area with a diameter of about 22 cm.

### 4.2. Results and Analysis

The main outputs of the KENPAVE simulations were the tensile stresses and strains at selected positions, i.e., at the bottom of the asphalt courses (29 cm depth) and at the bottom of the intact subbase (54 cm depth).

For the asphalt courses, the fatigue resistance (*N_f_AC_*) was then calculated as the sum of the number of traffic loads (i.e., 120 kN equivalent single axle loads (ESALs)) necessary for the crack initiation (*N_0_*) and the number of traffic loads (i.e., 120 kN ESALs) necessary for the propagation of the crack from the bottom of the asphalt courses to the pavement surface (Δ*N_0_*). *N_0_* was calculated according to the relationship proposed by Verstraeten et al. [43]:

(3) $$\log N_{0}=6+4.7619\cdot \left[\log\left(\lambda \cdot \frac{V_{b}}{V_{b}+V_{v}}\right)-\log\varepsilon_{t}\right]$$

where *ε_t_* is the tensile strain at the bottom of the asphalt courses (i.e., at the bottom of the base course) [με]; *V_b_* and *V_v_* are, respectively, the percentage of bitumen by mixture volume [%] and the air void content [%] in the base course mixture; *λ* is a coefficient that depends on the bitumen type, which was set as equal to the suggested value of 1.25·10^−4^ (for both HMA and WMA).

Instead, Δ*N_0_* was calculated according to the relationship proposed by Marchionna et al. [44] for typical Italian motorway pavements, which corresponds to a percentage of cracking on the pavement surface equal to 10%:

(4) $$\Delta N_{0}=\left(E^{\alpha^{\prime }}\cdot \sigma^{\beta^{\prime }}\cdot 10^{\gamma^{\prime }}\right)\cdot \left(1.373\cdot e^{-1.089\cdot n}\cdot h^{\left(-0.152+0.476\cdot n\right)}\right)$$

where *h* is the thickness of the dense-graded asphalt courses [cm]; *E* is the representative stiffness modulus of the dense-graded asphalt courses [kg/cm^2^], calculated as the weighted average of the stiffness of the binder and base courses; *σ* is the tensile stress at the bottom of the asphalt courses (i.e., at the bottom of the base course) [kg/cm^2^]; *n* is a parameter that mainly depends on the type of bitumen, which was set equal to 4.5 (typical value for mixtures containing polymer-modified bitumen); *α’*, *β’* and *γ’* are parameters whose expressions are a function of *n*, as follows: $\alpha^{\prime }=\left(2.43683\cdot n\right)/5$, $\beta^{\prime }=\left(-3.28354\cdot n\right)/5$, $\gamma^{\prime }=\left[\frac{-2.24181\cdot n}{5}\right]+0.847\cdot \left(1-\frac{n}{5}\right)$. It is worth noting that the contribution of the OGFC against crack propagation was conservatively neglected, given the relatively low cracking resistance that is typical of open-graded mixtures.

For the cold recycled subbase, the fatigue resistance (*N_f_S_*) was instead calculated as the number of traffic loads (i.e., 120 kN ESALs) that leads to cracking (i.e., to the beginning of the granular-like stage), according to Liebenberg and Visser [39]:

(5) $$N_{f\_S}=10^{7.92-1.28\cdot \left(\frac{\varepsilon_{t}}{\varepsilon_{b}}\right)}$$

where *ε_t_* is the tensile strain at the bottom of the cold recycled course [με]; *ε_b_* is the maximum admissible strain [με], which depends on the composition of the mixture. For the typical aggregate composition and bitumen content of Italian cold recycled subbases, *ε_b_* can be assumed to be equal to 230 με.

Afterwards, for each season (winter, spring, summer and autumn), the actual fatigue resistance of the pavement (*N_tot_*) was calculated through the following equation:

(6) $$N_{tot}=N_{f\_S}+N_{f\_AC\_CS}\cdot \frac{N_{f\_AC\_IS}-N_{f\_S}}{N_{f\_AC\_IS}}$$

where *N_f_S_* is the fatigue resistance of the cold recycled course; *N_f_AC_IS_* is the fatigue resistance of the asphalt courses in the case of intact subbase; *N_f_AC_CS_* is the fatigue resistance of the asphalt courses in the case of cracked subbase. In other words, such an equation expresses the actual fatigue resistance of the pavement as the sum of the fatigue resistance of the cold recycled course and the fatigue resistance of the asphalt courses in the case of cracked subbase, the latter multiplied by a correction factor (<1) that quantifies the portion of fatigue resistance still available for the asphalt courses after the subbase cracking.

Finally, the fatigue resistances related to the different seasons were combined based on Miner’s law on cumulative fatigue damage [45].

The outcomes of the KENPAVE simulations, i.e., the service life in terms of bottom-up cracking, are summarized in Table 3. It can be noted that, despite the differences in the properties of the materials (see Section 3), the fatigue resistance of the two trial sections is very similar and only slightly higher for the WMA section with respect to the HMA section. Considering the field trial’s annual traffic (8.5 million ESALs), a fatigue life of about 13 years and 11 years was obtained for the WMA section and the HMA section, respectively. Specifically, according to Equation (5), the cold recycled subbase remains intact for about 10 years for both sections. After the subbase cracking, both pavements have a remaining fatigue life of a few years.

## 5. FlexPAVE Simulations

### 5.1. Input Data

Eight different FlexPAVE simulations were performed, as summarized in Table 4. The code used to identify the simulations is as follows: the first part of the code indicates the analyzed trial section (HMA or WMA); the second part of the code indicates whether the subbase was considered intact (IS) or cracked (CS) in the simulation; the final part of the code indicates whether the simulation considered only stresses and strains induced by traffic loads (F) or also those induced by daily temperature variations (F + T).

**Figure 6:**
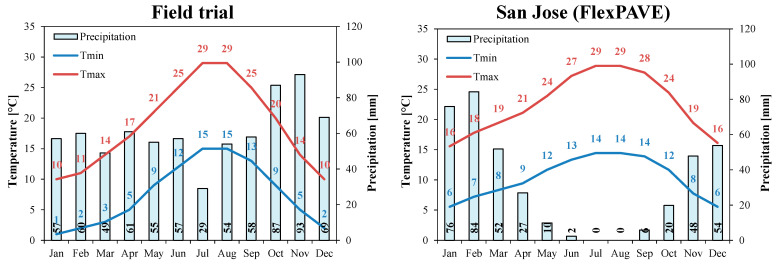
Comparison of the climatic conditions for the location of the field trial and San Jose (data available in FlexPAVE).

As compared to the KENPAVE simulations, the main differences in the input data were the properties assigned to the asphalt materials and the modeling of the climatic conditions. As reported in Table 4, in all cases, the OGFC was modeled based on the properties of the OG mixture (Figure 5), whereas the properties assigned to the binder and base courses were those determined from the laboratory tests carried out on the cores extracted from the trial sections (Figure 4). In terms of climatic conditions (annual temperatures and precipitations), Figure 6 shows that the location of the field trial is broadly comparable to San Jose (California), whose detailed climatic data (one-year daily temperature variations) are available in the FlexPAVE database.

The other inputs were analogous to the KENPAVE simulations. The subbase was modeled as a linear elastic layer with a stiffness modulus of 1200 MPa in intact conditions (as emerged from FWD tests carried out on the pavement [25]) and 400 MPa in cracked conditions (typical value for granular materials), whereas the subgrade was modeled as a linear elastic layer with a stiffness modulus of 100 MPa (based on the available FWD data [25]). As for the traffic loads, an annual traffic of 8.5 million 120 kN ESALs was considered. The traffic speed and the tire inflation pressure were equal, respectively, to 90 km/h and 800 kPa to assess the damage caused by motorway heavy traffic, and the tire–pavement contact area was assumed to be circular.

All simulations analyzed a period of 30 years, which is the maximum timespan that can be examined with FlexPAVE.

### 5.2. Results and Analysis

The main outputs of the FlexPAVE simulations were the damage contours, representing the distribution of the damage factor within the pavement (specifically, the asphalt courses). The damage factor is defined as the ratio of the current number of cycles (*N*) to the number of cycles at failure (*N_f_*) and thus ranges from 0 to 1, with 1 indicating a completely cracked asphalt element.

**Figure 7:**
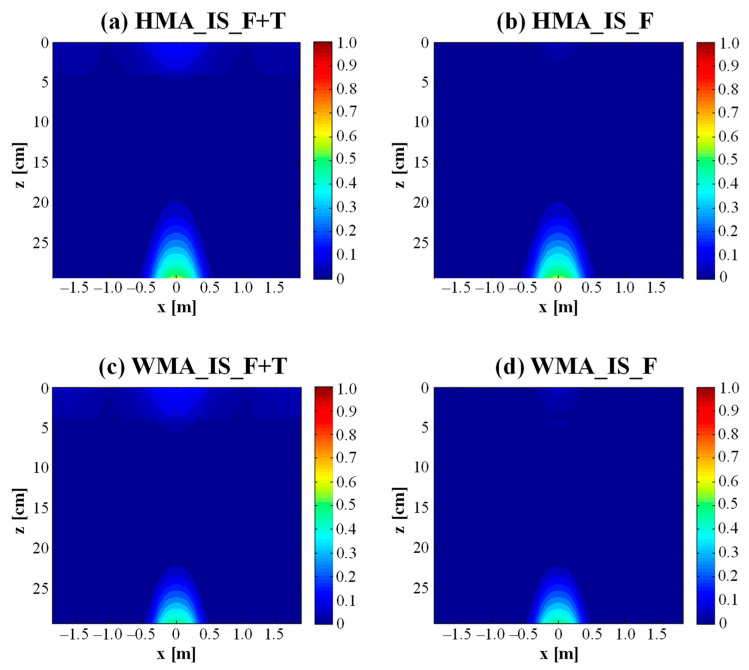
Damage contours in the case of intact subbase after 30 years.

Figure 7 presents the damage contours for the two trial sections after 30 years in the case of intact subbase. Figure 7a shows that, when considering both the stresses and strains induced by traffic loads and daily temperature variations, the HMA section exhibits a certain amount of damage at the bottom of the asphalt courses (bottom–up cracking), which involves about 9 cm of asphalt concrete. A limited amount of damage can be also observed in the upper 5 cm of the pavement (top–down cracking). The latter is mainly ascribable to thermal effects, since there is almost no damage in the upper part of the pavement when only the stresses and strains induced by traffic loads are considered (Figure 7b). As compared to the HMA section, the WMA section exhibits a lower amount of damage at the bottom of the asphalt courses, which affects about 6 ÷ 7 cm of asphalt concrete (Figure 7c). Limited top–down cracking, involving the upper 5 ÷ 6 cm of the pavement, is present also in this case (Figure 7c). The comparison between Figure 7c (stresses and strains induced by traffic plus thermal stresses and strains) and Figure 7d (only stresses and strains induced by traffic) indicates that the damage in the upper part of the pavement is mainly due to thermal effects, analogously to the HMA section.

**Figure 8:**
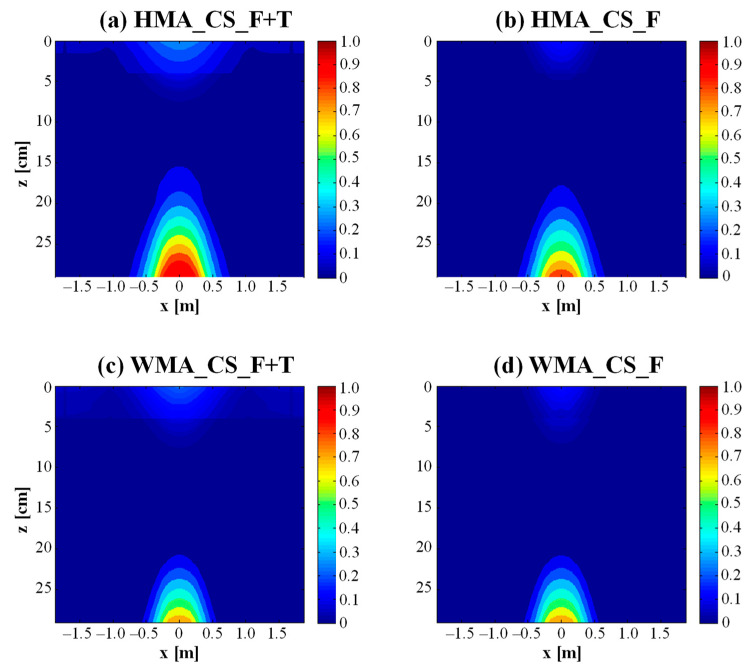
Damage contours in the case of cracked subbase after 30 years.

Figure 8 shows the damage contours obtained in the case of cracked subbase. As expected, in each case, the level and extent of damage are higher with respect to the corresponding simulation with intact subbase in Figure 7. From Figure 8a, it can be observed that, if the subbase has granular-like behavior, bottom–up cracking and top–down cracking affect, respectively, about 14 cm and 8 cm of asphalt concrete in the case of the HMA section. If the thermal effects are removed (Figure 8b), it can be noted that the amount of damage in the upper part of the pavement is significantly reduced. Without the thermal effects, even the level and extent of damage at the bottom of the asphalt courses appear to be reduced (Figure 8b). Analogously to the condition of intact subbase, the WMA section clearly exhibits a lower amount and extent of damage with respect to the HMA section (see Figure 8c vs. Figure 8a). The areas affected by bottom–up cracking and top–down cracking are both about 8 cm thick (Figure 8c). Moreover, for the WMA section, thermal damage mainly affects the upper part of the pavement, whereas it has a negligible effect on bottom–up cracking (see Figure 8d vs. Figure 8c).

Based on the damage contours, FlexPAVE also calculates the percentage of damage (*%Damage*) on the pavement cross-section, considering a reference area below the traffic load according to Equation (7) [23]:

(7) $$\%Damage=\frac{\sum_{i=1}^{M}{\left(N/N_{f}\right)}_{i}\cdot A_{i}}{\sum_{i=1}^{M}A_{i}}$$

where *i* indicates the nodal point number in the finite element mesh; *M* is the total number of nodal points in the finite element mesh; *A_i_* is the area belonging to nodal point *i* in the finite element mesh. Therefore, the evolution of *%Damage* over the analyzed timespan of 30 years was also obtained from the simulations.

**Figure 9:**
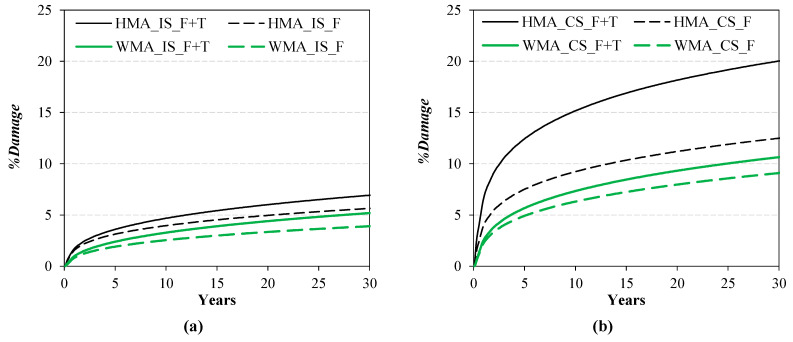
Damage evolution on the pavement cross-section over 30 years: (**a**) intact subbase condition, (**b**) cracked subbase condition.

**Figure 10:**
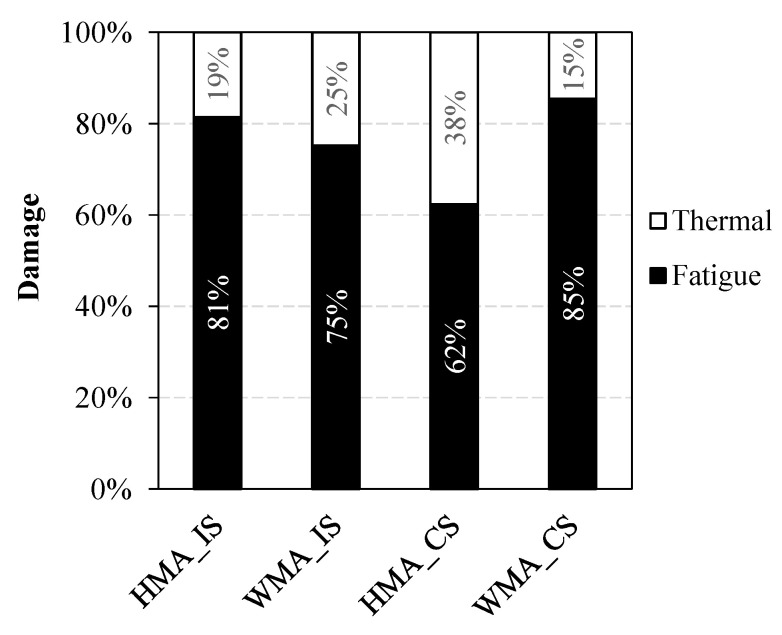
Influence of fatigue damage and thermal damage over the total damage obtained after 30 years.

Figure 9a,b show the evolution of *%Damage* over 30 years in the case of intact subbase and cracked subbase, respectively. From Figure 9a (intact subbase), it can be noted that, after 30 years, the percentage of damage induced by traffic loads plus thermal effects is about 7% for the HMA section and 5% for the WMA section. For both trial sections, 75 ÷ 80% of such damage is due to the traffic loads (i.e., pure fatigue damage), whereas the remaining 20 ÷ 25% of such damage is due to thermal effects (Figure 10) and is mainly concentrated in the upper part of the pavement, as discussed above. Figure 9b shows that, in all cases, *%Damage* is higher in the condition of cracked subbase than in the condition of intact subbase, as already observed from the comparison between Figure 7 and Figure 8. Under this condition, the percentage of damage induced by traffic loads plus thermal effects after 30 years is about 20% for the HMA section and 10% for the WMA section. For the WMA section, 85% of such damage is due to the traffic loads, whereas only 15% of such damage, concentrated in the upper part of the pavement, is due to thermal effects (Figure 10). Conversely, for the HMA section, the influence of thermal damage over the total damage is higher (38%, see Figure 10). In fact, from Figure 8a,b, it was already observed that thermal damage contributes both to top–down cracking and bottom–up cracking for the HMA section with cracked subbase.

The percentage of damage on the cross-section was then converted into the percentage of cracking on the pavement surface (*%Cracking*) by using the preliminary sigmoidal transfer function proposed by Wang et al. [46] based on the analysis of a variety of pavements with different characteristics and materials:

(8) $$\%Cracking=\frac{50}{1+C_{f1}\cdot \exp\left[C_{f2}\cdot \left(\log C_{f3}-\log\%Damage\right)\right]}$$

where *C_f_*_1_, *C_f_*_2_ and *C_f_*_3_ are calibration factors whose values are 0.342, 13.97 and 16.38, respectively; 50 is the maximum *%Cracking*, which corresponds to the complete fatigue cracking of the two wheel paths within the lane.

**Figure 11:**
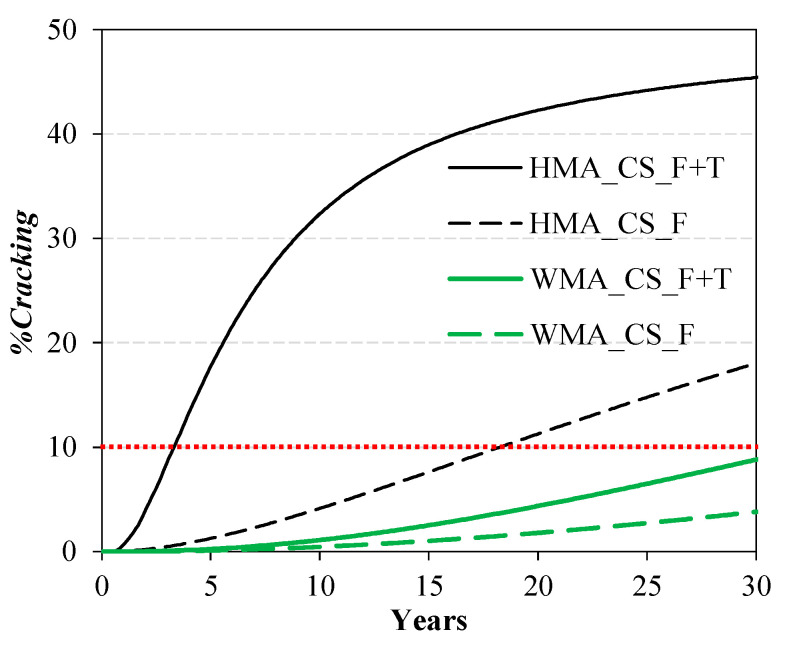
Cracking evolution on the pavement surface over 30 years with cracked subbase.

Figure 11 shows the evolution of *%Cracking* over 30 years in the case of cracked subbase. After 30 years, the predicted *%Cracking* is about 45% for the HMA section and slightly lower than 10% for the WMA section (considering both the damage induced by traffic loads and thermal damage). The evolution of *%Cracking* in the case of intact subbase is not shown, because, under these conditions, *%Cracking* remains lower than 1% in all cases even after 30 years. In fact, according to the considered sigmoidal transfer function (Equation (8)), when *%Damage* is lower than 10%, the corresponding *%Cracking* is lower than 5%. Conversely, when *%Damage* is higher than 10%, *%Cracking* increases dramatically. This feature of the transfer function is due to the fact that fatigue cracking is not observed on the pavement surface while fatigue damage is still growing within the asphalt courses [35,46].

For a better comparison with the KENPAVE results, a percentage of cracking of 10% on the pavement surface was considered also in this case to define the service life of the pavement (analogously to Equation (4)). The results regarding the condition of cracked subbase are summarized in Table 5. Considering both traffic-induced damage and thermal damage, the HMA section reaches the end of its service life after just 3 years, whereas the service life of the WMA section is much longer (around 30 years). However, it should be noted that KENPAVE does not take into account thermal stresses and strains, because a constant pavement temperature is considered in each season. Therefore, for a more realistic comparison with the KENPAVE results, the outcomes of the simulations without thermal damage should be considered. In this case, the service life of the HMA and WMA sections would be equal to 18 years and greater than 30 years, respectively (Table 5). Conversely, all simulations with intact subbase would lead to a service life much longer than 30 years (*%Cracking* lower than 1% after 30 years).

In this regard, it is worth noting that the fatigue lives obtained from the KENPAVE simulations in the case of intact and cracked subbases were properly combined to determine the actual fatigue resistance of the pavement (Equation (6)), i.e., broadly simulating the actual evolution of the subbase behavior from a bound material to a granular-like material during its in-service life. Therefore, a fairer comparison of the FlexPAVE results with the KENPAVE results should consider a service life that is intermediate between the case of intact subbase and the case of cracked subbase (in the presence of only traffic-induced damage).

Although the FlexPAVE predictions in terms of *%Cracking* may not be entirely realistic and need to be validated based on field observations, the results presented above clearly show that the expected service life of the WMA section is much longer than that of the HMA section. On the contrary, the KENPAVE simulations predict a similar service life for the two sections (see Table 3). In the KENPAVE simulations, the asphalt courses are modeled as linear elastic materials, and their mechanical properties are quantified mainly through the stiffness modulus. Consequently, KENPAVE is not able to differentiate two pavements that have a similar structure and comparable stiffness moduli (such as in the case of interest, see Table 2). FlexPAVE instead also considers the damage properties, which can be significantly different even for materials with comparable stiffness. In the case of interest, the damage properties of the WMA mixtures are better than those of the HMA mixtures (see Figure 4), which leads to a longer service life for the WMA section compared to the reference HMA section. In addition, from the FlexPAVE outcomes, it is evident that the thermal damage (which is basically ignored in the elastic simulations) cannot be neglected, as it can amount up to 40% of the total damage (see Figure 10). These findings highlight that the traditional elastic approach on which KENPAVE is based does not allow catching the actual contribution to the pavement service life provided by high-performance non-standard materials. Therefore, more advanced design tools should be used in order to predict the long-term behavior of the pavement more accurately, such as FlexPAVE.

## 6. Conclusions

The objective of this study was to predict the long-term performance of an existing warm recycled motorway pavement and the corresponding hot recycled pavement, both constructed in 2016 in central Italy. Cores were taken from the two pavements after about six years of in-service life. The binder and base courses were investigated through dynamic modulus and cyclic fatigue tests, based on the S-VECD testing approach. The properties of the materials determined from these tests were used to predict the service life of the two pavements using KENPAVE and FlexPAVE software.

According to the FlexPAVE results, the expected service life of the WMA pavement is much longer than that of the HMA pavement, as a result of the better damage properties of the WMA mixtures with respect to the HMA mixtures. On the contrary, the KENPAVE simulations predicted a similar service life for the two pavements. In fact, in the KENPAVE simulations, the asphalt courses are modeled as linear elastic materials, and their mechanical properties are quantified only through the stiffness modulus. Moreover, the thermal damage induced by the daily temperature variations is basically neglected in the elastic simulations, whereas it is fully taken into account by FlexPAVE. As a consequence, KENPAVE is able to differentiate only pavements with significant differences in the pavement structure (i.e., stratigraphy) and/or in the stiffness of the layers.

These findings indicate that warm recycled pavements can have better long-term performance and longer service life than conventional hot recycled pavements. Such promising results add up to the remarkable environmental benefits and energy savings provided by warm recycled pavements, further encouraging their application. Moreover, the findings of this study also highlight that the traditional elastic-based design method does not allow catching the actual contribution to the pavement service life provided by high-performance non-standard materials, suggesting the need to use more advanced design tools (such as FlexPAVE) for this purpose.

As a possible future work, the FlexPAVE predictions obtained in this study (especially those deriving from the application of the transfer function between *%Damage* and *%Cracking*) should be calibrated based on the actual pavement performance, which will be possible by continuing to monitor the pavements in question over time. Specifically, periodical investigations of the trial sections, including non-destructive testing with FWD and TSD (traffic speed deflectometer), as well as laboratory testing on extracted cores, are already planned to collect more data on the field behavior of warm recycled motorway pavements and to confirm/deny the predicted performance. Moreover, the predictions presented in this paper can also be improved by testing the cold recycled subbase in the laboratory in order to model this course as an asphalt-treated material.

## Figures and Tables

**Table 1:** Air void content of the mixtures.

Mixture	Air Void Content [%]
HMA-BINDER	4.7
WMA-BINDER	4.1
HMA-BASE	6.1
WMA-BASE	3.9

**Table 2:** Stiffness moduli considered in the KENPAVE simulations (values in MPa).

Course	Winter (T_AC_ = 13 °C)	Spring (T_AC_ = 18.4 °C)	Summer (T_AC_ = 32.4 °C)	Autumn (T_AC_ = 22.5 °C)
OGFC (4 cm)	8422	7025	3752	6002
HMA-BINDER (10 cm)	16,252	13,449	6449	11,313
WMA-BINDER (10 cm)	15,794	12,758	5611	10,491
HMA-BASE (15 cm)	13,370	10,774	4804	8871
WMA-BASE (15 cm)	16,188	13,321	6414	11,174
Intact subbase (25 cm)	1200	1200	1200	1200
Cracked subbase (25 cm)	400	400	400	400
Subgrade	100	100	100	100

**Table 3:** Outcomes of the KENPAVE simulations.

Trial Section	Fatigue Resistance [120 kN ESAL]	Annual Traffic [120 kN ESAL]	Fatigue Life [Years]
HMA	9.25 × 10^7^	8.50 × 10^6^	11
WMA	1.07 × 10^8^	8.50 × 10^6^	13

**Table 4:** Material properties considered in the FlexPAVE simulations.

Simulation	OGFC (4 cm)	Binder (10 cm)	Base (15 cm)	Subbase (25 cm)	Subgrade
HMA_IS_F + T	OG	HMA-BINDER	HMA-BASE	*E* = 1200 MPa	*E* = 100 MPa
HMA_IS_F	OG	HMA-BINDER	HMA-BASE	*E* = 1200 MPa	*E* = 100 MPa
WMA_IS_F + T	OG	WMA-BINDER	WMA-BASE	*E* = 1200 MPa	*E* = 100 MPa
WMA_IS_F	OG	WMA-BINDER	WMA-BASE	*E* = 1200 MPa	*E* = 100 MPa
HMA_CS_F + T	OG	HMA-BINDER	HMA-BASE	*E* = 400 MPa	*E* = 100 MPa
HMA_CS_F	OG	HMA-BINDER	HMA-BASE	*E* = 400 MPa	*E* = 100 MPa
WMA_CS_F + T	OG	WMA-BINDER	WMA-BASE	*E* = 400 MPa	*E* = 100 MPa
WMA_CS_F	OG	WMA-BINDER	WMA-BASE	*E* = 400 MPa	*E* = 100 MPa

**Table 5:** Outcomes of the FlexPAVE simulations in the case of cracked subbase.

Simulation	Service Life [Years]
HMA_CS_F + T	3
HMA_CS_F	18
WMA_CS_F + T	≈30
WMA_CS_F	>30

## Data Availability

The data presented in this study are available upon request from the corresponding author.
